# O Eletrocardiograma na População Pediátrica no Século XXI. Como Continuar Evoluindo após 135 Anos de História da Descoberta do Método

**DOI:** 10.36660/abc.20220715

**Published:** 2022-11-01

**Authors:** Rogerio Braga Andalaft

**Affiliations:** 1 Instituto Dante Pazzanese de Cardiologia – Eletrofisiologia São Paulo SP Brasil Instituto Dante Pazzanese de Cardiologia – Eletrofisiologia, São Paulo, SP – Brasil; 2 Hospital Israelita Albert Einstein São Paulo SP Brasil Hospital Israelita Albert Einstein, São Paulo, SP – Brasil

**Keywords:** Eletrocardiograma, Pediatria, Crianças, Arritmias

O conhecimento do eletrocardiograma permanece em constante evolução na prática médica. As novas interfaces de correlação do eletrocardiograma com as imagens cardíacas e alterações funcionais do coração propiciam o aumento da aplicabilidade do método. Entretanto, em crianças e adolescentes, a constante mudança gerada pelo desenvolvimento corporal e funcional destes jovens, muitas vezes geram dúvidas na interpretação dos traçados eletrocardiográficos mesmo nos profissionais mais experientes. Recentemente, dados importantes sobre a interpretação do eletrocardiograma (ECG) foram amplamente revisados nas IV Diretrizes da Sociedade Brasileira de Cardiologia sobre Análise e Emissão de Laudos Eletrocardiográficos publicada em 2022.^1^

Mas qual é o papel do eletrocardiograma, em especial aplicado à pediatria, no século XXI? O que é normal para a criança? Qual é a prevalência das alterações do eletrocardiograma em assintomáticos?

Um estudo nacional utilizando o sistema de tele ECG na população pediátrica de 0 a 10 anos assintomáticos evidencia a presença de alterações eletrocardiográficas com possibilidade de repercussão clínica. Foram avaliadas 3.139 crianças entre 0 e 10 anos assintomáticas e observamos a presença de bloqueios atrioventriculares em 0,41% e 0,44% de síndrome de pré-excitação ventricular. O intervalo QTC prolongado foi observado em 0,35% da população da população estudada.^2^ Durante o Congresso Europeu de Cardiologia em 2022 Fuziy et al. apresentaram dados de mais de 11.000 adolescentes assintomáticos traçando o perfil eletrocardiográfico destes jovens. Foi observado 0,5% de arritmias cardíacas sendo em sua maioria arritmias de origem atrial. Alterações eletrocardiográficas com risco de arritmias graves foram 0,13% de pré-excitação ventricular e alterações de repolarizacão incluindo QT prolongado em 1,8% da população.^3^

O ECG também é fundamental durante a avaliação de emergência da criança nas unidades de pronto atendimento. Nestes casos a identificação correta de um traçado pode permitir o tratamento adequado e melhores resultados terapêuticos.^4^ Em nosso meio, a Diretriz Brasileira de Arritmias Cardíacas em Crianças e Cardiopatias Congênitas fornece um guia fundamental para abordagem dos distúrbios do ritmo nesta população.^5^

Dados da tabela de Davignon, principalmente sobre a FC média para cada faixa etária, podem estar certamente superestimados devido às técnicas de fixação dos eletrodos, choro, estresse de outra origem ou condições clínicas adversas que possam motivar a realização do ECG. A utilização da tabela de Davignon como referência dos ECG normais de crianças é baseada em levantamento de 2.141 crianças brancas sem alterações sintomáticas ou estruturais realizado na cidade de Quebec, no Canadá, publicado em 1979. Assim, traçar parâmetros de normalidade em crianças de outras etnias e fruto de miscigenação, podem evidenciar diferenças em relação aos relatos iniciais de Davignon.

A avaliação eletrocardiográfica de rotina assim como o teste da saturação em neonatos poderia ser utilizada de rotina nos primeiros meses de vida com o intuito de detectar precocemente alterações eletrocardiográficas envolvidas na síndrome de morte súbita no leito.

O uso de dispositivos (relógios e celulares) para detectar alterações do ritmo cardíaco nestes jovens também vem ganhando volume na produção científica mundial e podem ser aplicados à diferentes faixas etárias, incluindo neonatos. As alterações de ritmo baseados na análise do complexo QRS, mas não na onda P, não apresentam diferenças em relação ao ECG para análise de ritmo com uma factibilidade de realização de mais de 94%.^6^ As diferentes modalidades diagnósticas que utilizam o ECG como base (Holter, teste ergométrico, monitor de eventos, teste de inclinação e estudo eletrofisiológico, por exemplo) estão em constate ampliação de indicações na população pediátrica e nas cardiopatias congênitas.^7,8^

Apesar da extrema importância da abordagem clínica pura, esta muitas vezes não permite ao médico descartar alterações fenotípicas elétricas que podem resultar em eventos cardiovasculares na infância e adolescência.^9^ Realizar um ECG como rotina na prática médica permite identificar precocemente grande parte das canalopatias e também descartar alterações que podem se agravar durante a prática esportiva, e atividades adrenérgicas que podem colocar a vida destes indivíduos em risco. O ECG é um método de triagem de baixo custo e ainda permite a profilaxia de eventos súbitos, partindo do diagnóstico precoce, o que tem um forte impacto sobre a saúde de uma família ou uma comunidade.

Neste século, a utilização dos sistemas de telemedicina propicia o levantamento de grande número de exames em curto período e um retrato fidedigno dos padrões eletrocardiográficos normais e da densidade de alterações eletrocardiográficas assintomáticas na população pediátrica. O ECG, sem sombra de dúvida, é o método ideal de triagem em jovens para detecção de anormalidades elétricas e algumas alterações estruturais, pois possui rápida exequibilidade, baixo custo, amplo conhecimento técnico divulgado, e acessibilidade nos diversos pontos de nosso país. Ainda, o acesso a centros especializados em laudos eletrocardiográficos poderá detectar por meio da telemedicina alterações precoces em neonatos e lactentes, assim como condições de risco na avaliação pré-prática esportiva. Em um país de dimensões continentais onde 65% dos municípios de acesso mais remotos estão nas regiões norte e centro oeste do país, a telemedicina representa o encurtamento de distância e principalmente a possibilidade de diagnósticos mais precoces e ajustes terapêuticos mais adequados particularmente no que se refere as canalopatias na população pediátrica.

Frente ao exposto podemos concluir que após mais de 100 anos de existência o eletrocardiograma ainda não atingiu seu ápice, vem se expandindo em indicações e usos, mudando sua forma de análise e ampliando seu uso em populações especificas.

## References

[1] Samesima N, God EG, Kruse JCL, Leal MG, França FFAC, Pinho C (2022). Diretriz da Sociedade Brasileira de Cardiologia sobre a Análise e Emissão de Laudos Eletrocardiográficos – 2022. Arq Bras Cardiol.

[2] Andalaft R, Cerutti VB, Lervolino RL, Ragognete RG, Felicioni SP, Almeida C, Nogueira MF, Moreira DAR, França FFA (2011). Diagnósticos de ECG na população pediátrica com o uso de um sistema de tele ECG. Arq Bras Cardiol.

[3] Nogueira MF, Andalaft RB, Berbert GH (2022). Electrocardiographic Profile of Asymptomatic Adolescents by the TELE ECG System in Brazil: Analysis of 11058 Patients.

[4] Guimarães HP, Andalaft RB, Carvalho P, Costa FA, Correa DC, Caldeira P (2017). Suporte Avançado de Vida em Pediatria Manual do Profissional.

[5] Magalhães LP, Guimarães I, Melo SL, Mateo E, Andalaft RB, Xavier L (2016). Diretriz de Arritmias Cardíacas em Crianças e Cardiopatias Congênitas SOBRAC E DCC - CP. Arq Bras Cardiol.

[6] Kobel M, Kalden P, Michaelis A, Markel F, Mensch S, Weidenbach M (2022). Accuracy of the Apple Watch iECG in Children With and Without Congenital Heart Disease. Pediatr Cardiol.

[7] Andalaft R (2012). Utilização dos Métodos não Invasivos em Diagnósticos das Arritmias na Infância. Relampa.

[8] França FFAC, Andalaft R., Timerman A, Bertolami M, Ferreira JFM (2012). Manual de Cardiologia.

[9] Andalaft R, Timerman A, Sousa A (2014). Condutas Terapêuticas do Instituto Dante Pazzanese de Cardiologia.

