# Supplementary material for: O Eletrocardiograma na População Pediátrica no Século XXI. Como Continuar Evoluindo após 135 Anos de História da Descoberta do Método
Source: Arq Bras Cardiol. 2022 Nov 1;119(5):791–2. [Article in Portuguese] doi: 10.36660/abc.20220715 (PMC9750202; doi:10.36660/abc.20220715)
Supplement: Translation

# Translation: The Electrocardiogram in the Pediatric Population in the 21st Century. How to Keep Evolving after 135 Years of the Method Discovery History

**Authors:** Rogerio Braga Andalaft

**Affiliations:** 1 São Paulo SP Brazil Instituto Dante Pazzanese de Cardiologia – Eletrofisiologia, São Paulo, SP – Brazil; 2 São Paulo SP Brazil Hospital Israelita Albert Einstein, São Paulo, SP – Brazil

**Keywords:** Electrocardiogram, Pediatrics, Children, Arrhythmias

Knowledge of the electrocardiogram remains constantly evolving in medical practice. The new interfaces for electrocardiogram (ECG) correlation with cardiac images and heart functional changes increase the method's applicability. However, in children and adolescents, the constant change generated by these young people's body and functional development often generates doubts in the interpretation of electrocardiographic tracings, even in the most experienced professionals. Recently, important data on ECG interpretation were extensively reviewed in the IV Guidelines of the *Sociedade Brasileira de Cardiologia* on Analysis and Issuance of Electrocardiographic Reports published in 2022.^1^

But what is the role of the electrocardiogram, especially applied to pediatrics, in the 21^st^ century? What is normal for the child? What is the prevalence of electrocardiogram changes in asymptomatic patients?

A national study using the tele ECG system in the asymptomatic pediatric population aged 0 to 10 years evidences the presence of electrocardiographic alterations with the possibility of clinical repercussions. A total of 3,139 asymptomatic children aged between 0 and 10 years were evaluated, and we observed the presence of atrioventricular blocks in 0.41% and 0.44% of the ventricular pre-excitation syndrome. A prolonged QTC interval was observed in 0.35% of the population studied.^2^ During the European Congress of Cardiology in 2022, Fuziy et al. presented data from more than 11,000 asymptomatic adolescents tracing the electrocardiographic profile of these young people. It was observed 0.5% of cardiac arrhythmias, mostly atrial arrhythmias. Electrocardiographic changes with risk of serious arrhythmias were 0.13% of ventricular pre-excitation and repolarization changes, including prolonged QT in 1.8% of the population.^3^

The ECG is also essential during the emergency assessment of the child in emergency care units. In these cases, correct tracing identification can allow adequate treatment and better therapeutic results.^4^ In our country, the Brazilian Guideline on Cardiac Arrhythmias in Children and Congenital Heart Diseases provides a fundamental guide for addressing rhythm disorders in this population.^5^

Data from the Davignon table, mainly on the average HR for each age group, can certainly be overestimated due to electrode fixation techniques, crying, stress from another source or adverse clinical conditions that may motivate the performance of the ECG. The use of the Davignon table as a reference for normal ECGs in children is based on a survey of 2,141 white children without symptomatic or structural alterations in Quebec City, Canada, published in 1979. Thus, tracing parameters of normality in children of other ethnicities and the result of miscegenation may show differences concerning Davignon's initial reports.

Routine electrocardiographic assessment and saturation testing in neonates could be routinely used in the first months of life with the aim of early detection of electrocardiographic alterations involved in sudden death in bed syndrome.

The use of devices (watches and cell phones) to detect changes in heart rhythm in these young people has also been gaining volume in the world's scientific production and can be applied to different age groups, including neonates. Rhythm alterations based on the analysis of the QRS complex, but not on the P wave, do not show differences concerning the ECG for rhythm analysis, with the feasibility of performing more than 94%.^6^ The different diagnostic modalities that use the ECG as a basis (Holter, exercise test, event monitor, tilt test and electrophysiological study, for example) constantly expand their indications in the pediatric population and congenital heart diseases.^7,8^

Despite the extreme importance of the pure clinical approach, it often does not allow the physician to rule out electrical phenotypic alterations that can result in cardiovascular events in childhood and adolescence.^9^ Performing an ECG as a routine in medical practice allows the early identification of most channelopathies and also discards alterations that can worsen during sports practice and adrenergic activities that can put the lives of these individuals at risk. The ECG is a low-cost screening method and allows for the prophylaxis of sudden events, starting with early diagnosis, which strongly impacts the health of a family or community.

In this century, telemedicine systems allow the survey of many exams in a short period and a reliable portrait of normal electrocardiographic patterns and the density of asymptomatic electrocardiographic changes in the pediatric population. Undoubtedly, the ECG is the ideal screening method for young people to detect electrical abnormalities and structural alterations, as it has rapid feasibility, low cost, extensive technical knowledge, and accessibility in different parts of our country. Furthermore, access to centers specialized in electrocardiographic reports can detect, through telemedicine, early changes in neonates and infants, as well as risk conditions in the pre-practice sports assessment. In a country of continental dimensions, where 65% of the most remote access municipalities are in the north and central west of the country, telemedicine represents the shortening of distance and the possibility of earlier diagnoses and more adequate therapeutic adjustments, particularly concerning channelopathies in the pediatric population.

Because of the above, we can conclude that after more than 100 years of existence, the electrocardiogram has not yet reached its peak; it has been expanding in indications and uses, changing its form of analysis and expanding its use in specific populations.
